# Investigation of SLM Process in Terms of Temperature Distribution and Melting Pool Size: Modeling and Experimental Approaches

**DOI:** 10.3390/ma12081272

**Published:** 2019-04-18

**Authors:** Md Jonaet Ansari, Dinh-Son Nguyen, Hong Seok Park

**Affiliations:** Department of Mechanical Engineering, University of Ulsan, Ulsan 44601, Korea; jonaetansari@gmail.com (M.J.A.); ngdison@gmail.com (D.-S.N.)

**Keywords:** Additive manufacturing, selective laser melting, volumetric heat source, thermal capillary effects, melt pool size

## Abstract

Selective laser melting (SLM) is an additive manufacturing (AM) technique that has the potential to produce almost any three-dimensional (3D) metallic part, even those with complicated shapes. Throughout the SLM process, the heat transfer characteristics of the metal powder plays a significant role in maintaining the product quality during 3D printing. Thus, it is crucial for 3D-printing manufacturers to determine the thermal behavior over the SLM process. However, it is a significant challenge to accurately determine the large temperature gradient and the melt pool size using only experiments. Therefore, the use of both experimental investigations and numerical analysis can assist in characterizing the temperature evaluation and the melt pool size in a more effective manner. In this study, 3D finite element analysis applying a moving volumetric Gaussian laser heat source was used to analyze the temperature profile on the powder bed and the resultant melt pool size throughout the SLM process. In the experiments, a TELOPS FAST-IR (M350) thermal imager was applied to determine the temperature profile of the melting pool and powder bed along the scanning direction during the SLM fabrication using Ti_6_Al_4_V powder. The numerically calculated results were compared with the experimentally determined temperature distribution. The comparison showed that the calculated peak temperature for single- and multi-track by the developed thermal model was in good agreement with the experiment results. Secondly, the developed model was verified by comparing the melting pool size for various laser powers and scanning speeds with the experimentally measured melting pool size from the published literature. The developed model could predict the melt pool width (with 2–5% error) and melt pool depth (with 5–6% error).

## 1. Introduction

Additive manufacturing (AM), widely familiar as 3D printing, is rapidly emerging as a new and disruptive manufacturing technology that offers the opportunity to manufacture complex, freeform three-dimensional (3D) metal parts with a 3D computer-aided design (CAD) design, and an AM printing machine [1]. Currently, AM has received increased attention from the aerospace, automotive, biomedical, and energy industries due to its benefits compared with conventional forming techniques. This technology acts an essential role in Industry 4.0 as the dominant technology that permits the cost-effective realization of the Internet of Things across various industrial applications. In recent years, various alloys such as stainless steel, titanium, aluminum, and nickel-based alloys are preferable for use in additive manufacturing technology. Ti_6_Al_4_V is the most popular alloy due to its lower density, elevated temperature properties, high strength-to-weight ratio, and good weldability characteristics [2,3,4,5].

Throughout the SLM process, the metal powder is continuously melted and fused by a high-intensity laser beam to fabricate high density, complicated metallic parts from a CAD model [6]. As a result, cross-sections of a certain zone are melted in layers, which are built up continuously to fabricate entire 3D products. Due to its unique process behaviors, SLM can be used to fabricate any intricate and high precision shape for metal parts, including elements with complicated porous shapes, which would be hard to produce with traditional manufacturing technologies [7,8].

Due to the continuous flow of energy through heat transfer in successive layers of the part, large temperature gradients are generated in the fabricated part. Consequently, it is essential to understand the inherent characteristics related to SLM processes (such as the temperature gradient and the melting pool size) before performing the actual printing process. This is because these characteristics influence the mechanical and physical properties as well as the overall quality of printed products. Moreover, the quality of the final SLM parts significantly depends on the appropriate selection of process parameters including laser power, scan speed, laser spot size, hatch distance, scanning pattern, and layer thickness. For that reason, it is always crucial to find out the optimal process parameters [9]. It is essential to develop a tool that can determine the temperature profile and melt pool size from both industrial and technical perspectives for optimizing the manufacturing processes and to control the consistent quality of the printed products.

The finite element (FE) technique is the most usually applied numerical approach for investigating the temperature profile and melt pool size in the SLM process. Shuai et al. [10] presented a mathematical model and established an FE method for determining the dynamic temperature distributions during the SLS process. Song et al. [4] performed a numerical analysis to predict the temperature field as well as experimental work to determine the relationship between the density, porosity, and laser scanning speed to identify better process parameters for the SLM process. Ma and Bin [11] proposed an FE analysis to determine the effect of two different laser moving strategies on the temperature distribution, residual stresses, and deformation during the selective laser sintering (SLS) process. Hussein et al. [9] developed an FE model that employed thermo-mechanical analysis for evaluating the development of temperature fields, melt pool size, and residual stress at various points on a single layer fabricated by the SLM process. Ali et al. [12] analyzed the influence of layer thickness on the thermal stress and mechanical properties of the SLM process for Ti_6_Al_4_V products by changing the applied laser power. Fischer et al. [13] conducted an experimental investigation to measure the peak temperature for the continuous wave and pulsed sintering process using an infrared camera. Dai and Shaw [14] presented an FE model to determine the effect of volume shrinkage associated with phase change during laser forming, and experimental work was performed to verify the presented model. Yadroitsev et al. [15] used a CCD camera for determining the highest temperature and the melt pool size of Ti_6_Al_4_V and also investigated the microstructure of SLM printed parts. Huang et al. [8] provided a detailed model of the temperature distribution and melt pool size that considered the volume shrinkage and linear energy density; this model was verified by comparing with published experimental results. Dilip et al. [3] experimentally measured the melt pool size and identified the relationship between porosity and energy density by changing the SLM process parameters. Lee and Zhang [16] presented a model of the macroscopic fluid and heat transfer incorporating Marangoni effects. They observed that Marangoni convection dramatically influenced the thermal behavior in the developed melt pool at the time of the SLM process. Ali et al. [17] presented a model that can predict the temperature distribution, melt pool width (with 14.5% error), melt pool depth (with 3% error), and the developed residual stress within the fabricated parts for various SLM process parameters. As noticed from this overview, 3D finite element models have been broadly accepted in cases of the SLM process. However, there have been few studies that used this technique to inspect the temperature evaluation and the developed melt pool size throughout the SLM process.

In this study, a 3D finite element (FE-Equations) model was initiated to analyze the thermal behavior and the developed melt pool size in a multi-track pattern during the SLM process. A moving volumetric Gaussian heat source was used with consideration of the temperature-dependent material properties and the phase transition of the Ti_6_Al_4_V alloy. Moreover, the variation of thermal behavior, and the developed melt pool size were investigated by applying various SLM process parameters. Finally, the corresponding experimental works were performed to validate the predicted results.

## 2. 3D Finite Element Modeling

**Figure 1:**
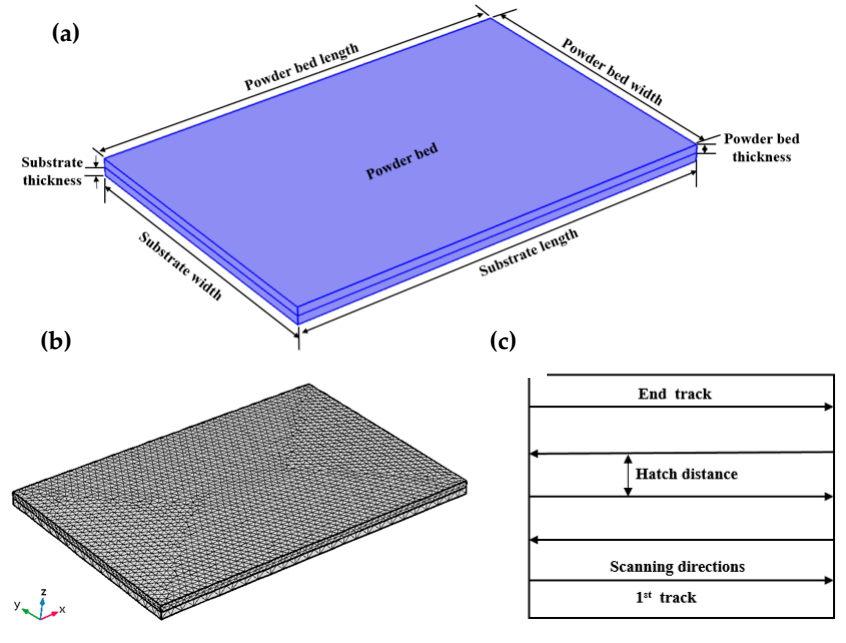
3D FE model of multi-track laser scanning throughout the SLM process. (**a**) Schematic of the designed geometry containing the substrate and powder bed, (**b**) 3D finite element mesh, and (**c**) scanning strategy.

A 3D thermal analysis model was developed using COMSOL Multiphysics Modeling Software (Version 5.4, COMSOL Ltd., Cambridge, UK) to predict the global temperature fields generated during laser irradiation. The 3D numerical model, mesh structure, and the SLM scanning patterns are shown in Figure 1. The dimensions of the modeled powder bed were a length of 10 mm, a width of 6.8 mm, and a thickness of 0.2 mm. A block with dimensions of 10 mm × 6.8 mm × 0.2 mm (length × width × thickness) was considered to be the substrate. To acquire the finest calculating efficiency within the smallest computational time, an extremely fine mesh was used for the powder bed, while a comparatively fine mesh has been used for the substrate. The numerical model consists of 30762 total domain elements, 9962 boundary elements, and 432 edge elements. The total computational time required for this presented simulation is about 9–10 h when using a workstation with an Intel Xeon CPU E5-2620 v2 @2.10GHz, RAM 32 GB (Intel, Santa Clara, CA, USA). The heat source moved in a bidirectional scanning direction. The process parameters that were applied in the FE analysis are listed in Table 1. The entire Ti_6_Al_4_V powder bed was presumed as a continuous and homogenous ambience. The coefficient of convective heat transfer among the powder bed and the circumstances were presumed to be a constant.

### 2.1. Governing Equation and Volumetric Heat Source

The thermal equilibrium is achieved based on the following transient 3D heat conduction equation, which can be expressed as [18]

(1) $$\rho C_{p}\frac{\partial x}{\partial y}+\rho C_{p}u\nabla T=\nabla \left(k\nabla T\right)+Q$$

in which *T* is the temperature, *ρ* is the density, *Сp* is the specific heat capacity, *k* is the thermal conductivity, *Q* is the absorbed heat, and *u* is the laser scanning speed.

Throughout the SLM process, the heat from the laser beam experiences numerous absorption and reflections across the Ti_6_Al_4_V powders. The applied laser energy is separated into three portions, including reflection, absorption, and transmission of power. Only the absorbed energy was used to melt the powders. The laser energy can travel a certain depth through the powder bed. Therefore, the heat transfer through the depth direction on the powder bed was also considered in this presented model. As presented by Li et al. [19], the Beer-Lambert attenuation law can be used to define the laser penetration in the depth direction, which is given as

(2) $$Q\left(x,y,z\right)=\frac{Q_{0}(x,y)}{\delta }\exp(-\frac{\left|z\right|}{\delta })$$

Here, *Q_0_* is the heat flux on the upper surface (W/m^2^), *δ* is the optical penetration depth for used material [20], *|z|* is the absolute value of the z-coordinate.

The distribution of surface heat flux *Q_0_* across the powder bed is presumed to be a Gaussian relationship, which can be mathematically represented as [13]

(3) $$Q_{0}\left(x,y\right)=\frac{2AP}{\pi R^{2}}\exp(-\frac{2({\left(x-ut\right)}^{2}+y^{2})}{R^{2}})$$

where *P* is the laser power, *A* is the laser energy absorption coefficient, and *R* is the resulting heat source radius at which the energy density is minimized to 1/e^2^ at the center of the laser spot. The laser scanning direction is included by replacing *x* with (*x-ut*). By replacing Equation (3) into Equation (2), the volumetric heat source is given as:

(4) $$Q\left(x,y,z\right)=\frac{2AP}{\pi \delta R^{2}}\exp(-\frac{2({\left(x-ut\right)}^{2}+y^{2})}{R^{2}})\exp(-\frac{\left|z\right|}{\delta })$$

Temperature-dependent thermal properties (*ρ, Сp, k*) of solid and liquid Ti_6_Al_4_V alloy were used in this study and were obtained from the published literature by Boivineau et al. [21], Heigel et al. [22], and Parry et al. [23]. The phase change behavior among the solid and liquid stages of a material can be input into Equation (1) with the following relationships [24]:

(5) $$\rho =\theta \rho_{solid}+(1-\theta )\rho_{liquid}$$

(6) $$C_{p}=\frac{1}{\rho }(\theta \rho_{solid}C_{p,solid}+(1-\theta )\rho_{liquid}C_{p,liquid})+L\frac{da}{dT}$$

(7) $$k=\theta k_{solid}+(1-\theta )k_{phase2}$$

(8) $$a=\frac{(1-\theta )\rho_{liquid}-\theta \rho_{solid}}{\theta \rho_{solid}+(1-\theta )\rho_{liquid}}$$

(9) $$\theta =\left\{\begin{array}{cc} 0, & if\;T\leq T_{S}\\ \frac{T-T_{L}}{T_{L}-T_{S}}, & if\;T_{S}<T<T_{L}\\ 1, & if\;T\geq T_{L} \end{array}\right\}$$

where *θ* is the phase fraction, *L* is the latent heat of the phase transfer from solid to liquid and *a* is the mass fraction, *T_L_* is the liquidus temperature and *T_S_* is the solidus temperature.

### 2.2. Fluid Flow Modeling

The large temperature gradient in the melting pool will cause a significant surface tension gradient, which creates Marangoni effects. These effects are also considered in this work and are given as [16]

(10) $$F^{Marangoni}=\nabla_{s}\gamma \;\begin{array}{cc} where & \gamma =\gamma_{0}+\frac{d\gamma }{dT} \end{array}\Delta T$$

where $\nabla_{s}$ denotes the surface gradient, *γ* is the surface tension, *γ_0_* is the surface tension at the liquidous point, *dγ/dT* is the surface tension gradient, and *ΔT* is the temperature difference.

To consider the flow behavior in the molten pool, the Navier-Stokes equations were used to model the laminar flow in the melt pool and are given as [16]

(11) $$\rho \frac{\partial u}{\partial t}+\rho (u.\nabla )u=\nabla .[-pI+\mu (\nabla u+{\left(\nabla u\right)}^{T})]+\rho g+F$$

(12) ρ∇.(u)=0

where *p* is the pressure, *µ* is the dynamic viscosity, *I* is the three-dimensional unity tensor, *ρg* is the gravity force, and *F* represents the other body forces, which are surface tension gradient-driven Marangoni forces already described in this work.

### 2.3. Initial and Boundary Conditions

The initial conditions of the finite element model include a uniform temperature field all over the powder bed before applying the heat source, which can be described as,

*T (x, y, z, 0)* = *T_0_ (x, y, z) for the whole domain at t = 0* (13)

where *T_0_ (x, y, z)* is the surrounding temperature and generally assumed as 293.15 K.

At the top surface of the developed model, heat transfer occurs among the powder bed, substrate, and their surroundings.

(14) $$-k\frac{\partial T}{\partial n}=h_{c}(T-T_{0})+\varepsilon \sigma (T^{4}-T_{0}^{4})$$

Here, the terms on the right side of the equation denote the heat loss owing to convection and radiation, successively. Furthermore, *n* denotes the normal direction of the surface, *h_c_* is the convective heat transfer coefficient, *ε* is the surface emissivity, and *σ* is the Stefan–Boltzmann constant.

To solve the mathematically derived problem, the FE method was developed by means of COMSOL Multiphysics in which two-ways coupling between heat transfer and fluid flow were established.

## 3. Materials and Experimental Methods

SLM experiments were performed using a SLM printer (MetalSys150, Winforsys co., Ltd., Yongin-si, South Korea) with a YLP-200-AC-Y11 IPG Ytterbium Fiber Laser (Winforsys co., Ltd., Yongin-si, South Korea) (highest laser power of 200 W). The chamber was occupied with argon protection gas to keep the oxygen level below 0.1%.

In this study, a commercial Ti-alloy (Ti_6_Al_4_V) powder provided by the (SLM Solutions Group AG, Lübeck, Germany), was used as a raw material with the resulting nominal chemical composition (wt.%): Ti-balance, Al-(5.5–6.50), V-(3.50–4.50), Fe-0.25, C-0.08, N-0.03, O-0.13, H-0.0125. The average powder size was about 23–60 μm.

**Figure 2:**
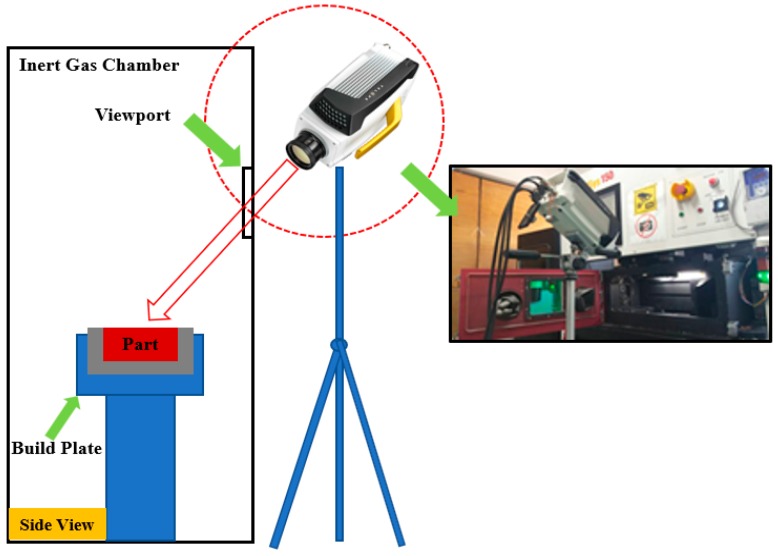
Schematic plot for experimental set up.

A TELOPS FAST-IR (M350) thermal camera (TELOPS, Quebec City, Canada) with a spatial resolution of 640 pixels × 512 pixels, and a maximum frame rate of 4980 Hz was employed to determine the temperature profile on the melt pool and the powder bed. The built part has dimensions of 10 × 6 × 0.03 mm (length × width × thickness) e.g., one layer. The camera was mounted on a tripod and fitted near to the view window, straightly focusing on the laser scanning area as presented in Figure 2. The process parameters for experiments were chosen as follows: laser powers (*P*): 120 W, 150 W; scan speeds (*u*): 750 mm/s, 1000 mm/s.

## 4. Numerical Model Validation

To confirm the thermal model and numerical approaches presented in this research, the developed model was initially compared with published experimental results.

**Figure 3:**
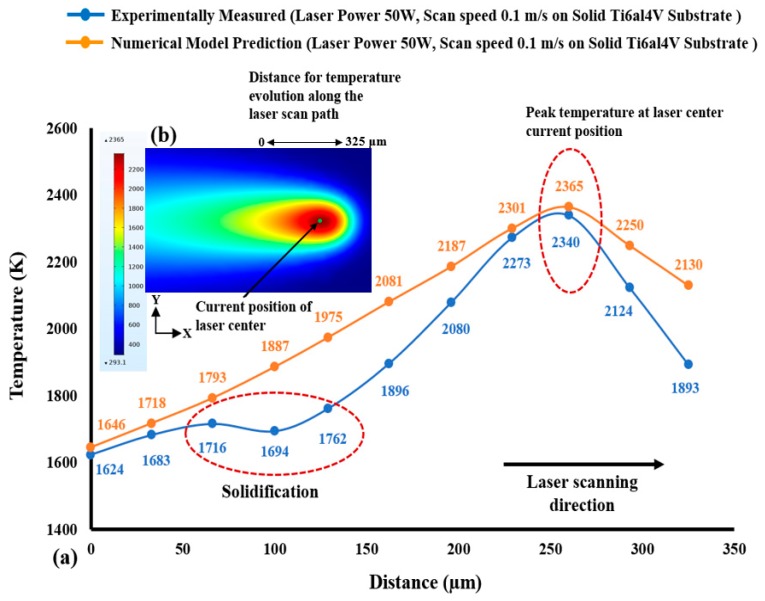
(**a**) Comparison of the FE model calculated temperature in the *xy*-plane along the laser moving path with the experimentally measured peak temperature along the laser scanning direction adapted from reference [15] and (**b**) Numerically predicted temperature distribution along the *xy*-direction considering a 325 µm laser scan path.

Yadroitsev et al. [15] experimentally measured the brightness temperature of the melt pool by using a laser power of 50 W and a scanning speed of 100 mm/s for Ti_6_Al_4_V alloy. Figure 3a illustrates the contrast of the calculated temperature field in the *xy*-plane across the laser moving path (presented in Figure 3b) with the experimentally measured peak temperature for the SLM of Ti_6_Al_4_V. Based on this comparison, the calculated peak temperature across the laser scanning direction concedes well with the trends in the experimentally determined temperature profile.

Fischer et al. [13] used a Raytheon infrared camera for determining the temperature distribution by applying a laser power of 3 W and a scanning speed of 1 mm/s.

**Figure 4:**
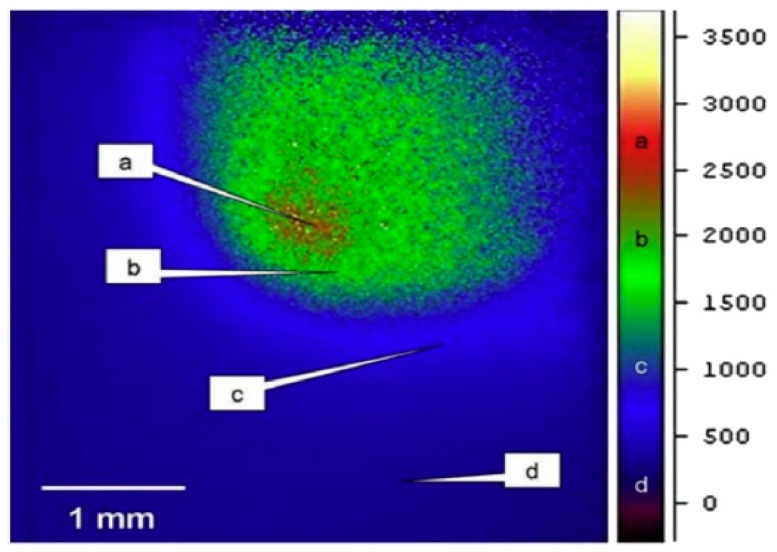
Experimentally measured average temperature profile throughout the laser sintering process at *P* = 3 W and *u* = 1 mm/s.

**Figure 5:**
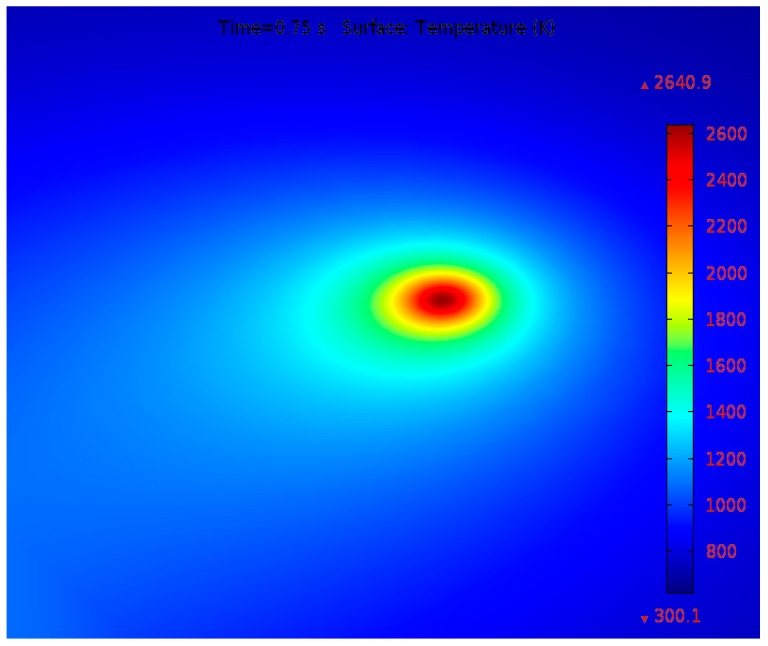
Numerically predicted surface temperature contours at *P* = 3 W and *u* = 1 mm/s.

Figure 4 demonstrates the temperature profile measured by Fischer et al. [13] and the peak temperature scale was in the range of 2500 K to 3000 K. Numerical analysis was conducted applying process parameters similar to those used by Fischer et al. [13] during their experiments. As demonstrated in Figure 5, the highest temperature after 0.75 s is about 2640.9 K which falls between the measured experimental values.

## 5. Results and Discussion

### 5.1. Temperature Distribution

During the SLM process, the temperature field on the powder bed deviates quickly with time and locations; these are critical issues for the printed product quality. The melting zone undergoes solidification in the wake of the heat source because of the moving laser beam and fast heat transmission from the melting zone to the surroundings. The solidification process starts once the temperature of the melt pool falls under the liquidus temperature. Afterwards it cools down to the ambient temperature for fabricating the complete products.

**Figure 6:**
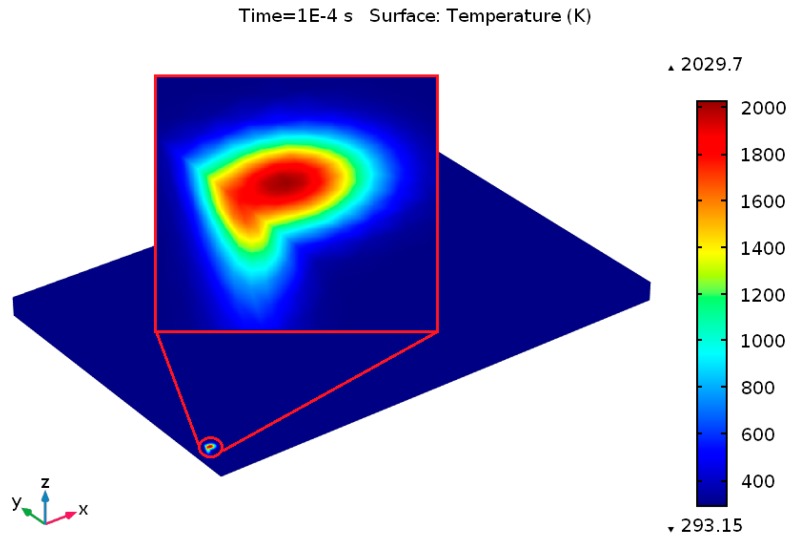
Temperature distribution at the start of laser scanning.

**Figure 7:**
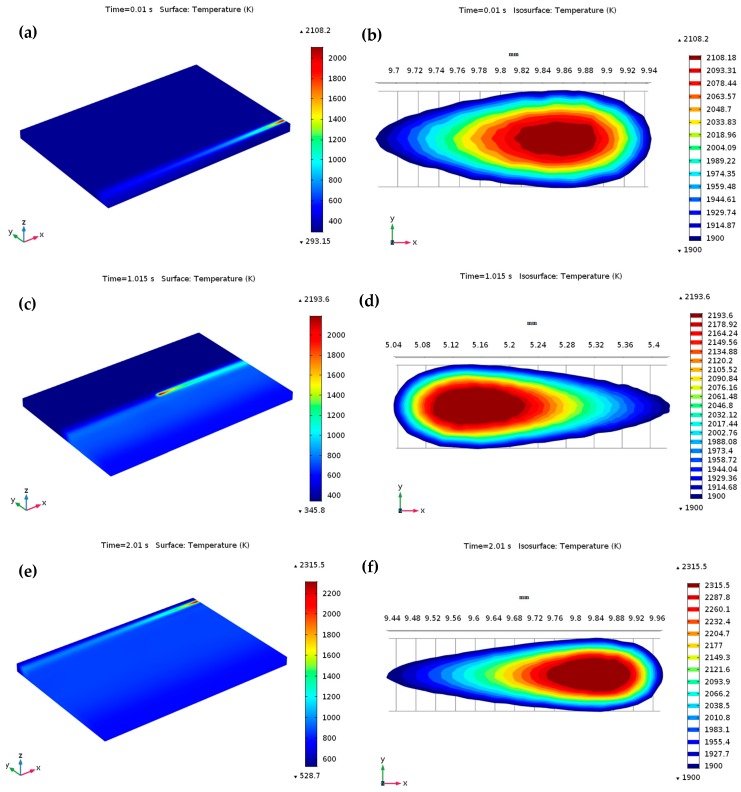
Temperature contours during the SLM process at *P* = 120 W and *u* = 1000 mm/s: (**a**) on the Ti_6_Al_4_V powder bed at the ending of the first scanning track at *t* = 0.01s and (**b**) isothermal contours around the melt pool at *t* = 0.01 s; (**c**) on the middle of Ti_6_Al_4_V powder bed at *t* = 1.015 s and (**d**) isothermal contours around the melt pool at *t* = 1.015 s; (**e**) on the Ti_6_Al_4_V powder bed at the ending of the last scanning track (after scanning a total of 201 tracks) at *t* = 2.01 s and (**f**) isothermal contours around the melt pool at *t* = 2.01 s.

Figure 6 illustrates the temperature contours at the start of laser moving, from which the peak temperature gradient in the laser spot region can be distinctly seen to the used 3D Gaussian heat source. The temperature of the powder bed rises quickly owing to the absorption of high energy irradiated from the heat source, initiating a melting pool in the powder bed once the temperature surpasses the liquidus point of Ti_6_Al_4_V (1928 K). Figure 7 illustrates the predicted temperature profiles and melt pool formation as the heat source reached various locations throughout the SLM process for *P* = 120 W and *u* = 1000 mm/s. Figure 7a demonstrates the temperature profiles at the final point of the first scanning track (at *t* = 0.01 s). At this point, the predicted highest temperature on the melt pool was about 2108.2 K, which exceeded the liquidus temperature of Ti_6_Al_4_V. Besides, the lowest temperature was only 293.15 K in most of the area of the powder bed and the substrate. The corresponding temperature contours and the melt pool region is presented in Figure 7b starting with the isothermal contours at 1900 K and going to the highest temperature of the melt pool. The melt pool size, as presented by the isothermal contours, provides a visual idea of the spatial energy distribution for a consistent heat source. The calculated temperature in the isothermal contours was larger than the liquidus temperature of Ti_6_Al_4_V. As a result, a little melt pool developed within this region. The length and width of the melt pool were approximately 237.6 μm and 90.7 μm, respectively. As the heat source arrived at the middle of the powder bed at *t* = 1.015 s, the maximum temperature of the melt pool increased to 2193.6 K in the center of the melt pool, as presented in Figure 7c. The melt pool length and width improved by approximately 356.4 μm and 110.2 μm, at a time of 1.015 s, as shown in Figure 7d. The heat source moved to its final position, and after that the heat source was no longer specified. Thus, only heat loss happens at this end position. At the ending of the final scanning track at *t* = 2.01 s, the predicted lowest and highest temperature of the powder bed raised to approximately 528.7 K and 2315.5 K, respectively, as presented in Figure 7e. Figure 7f represents the resulting melt pool length and width (approximately 512.2 μm and 137.8 μm) at a time of *t* = 2.01 s, which are larger than those at 0.01 s and 1.015 s. Therefore, the length of the melting pool increased more than the width of the melting pool as the laser irradiating time increased on the powder bed. For the specified numerical circumstances, the width of the melt pool at various positions was larger than the hatch distance (30 μm), which led to smooth melt tracks due to the development of a large enough melt pool between the adjacent tracks.

### 5.2. Variation of Temperature Distribution with Different Process Parameter

**Figure 8:**
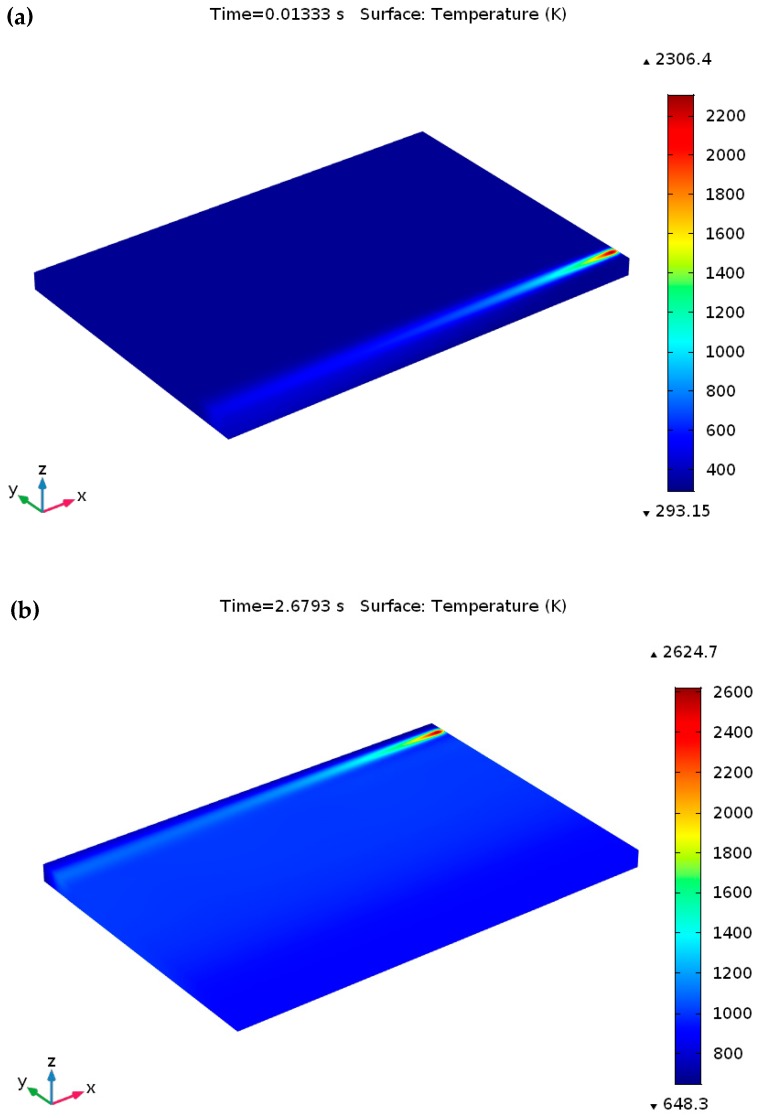
Temperature profiles throughout the SLM process at *P* =120 W and *u* = 750 mm/s. On the Ti_6_Al_4_V powder bed (**a**) at the ending of the first scanning track at *t* = 0.01333s and (**b**) at the ending of the final scanning track (after scanning a total of 201 tracks) at *t* = 2.6793s.

**Figure 9:**
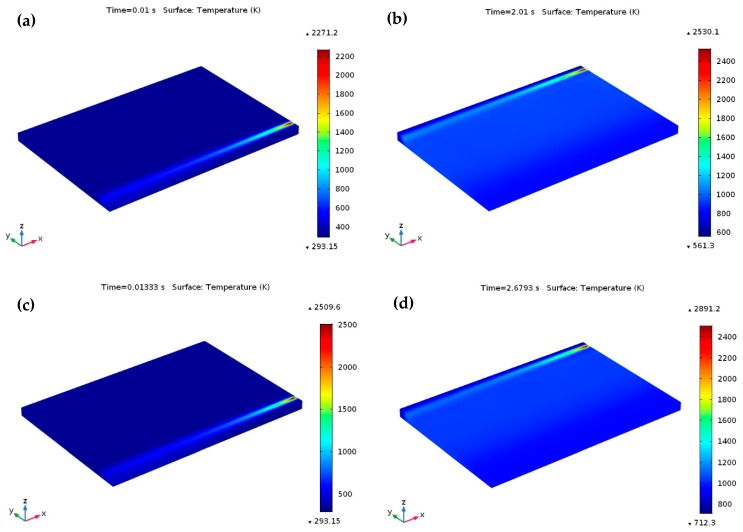
Temperature profiles throughout the SLM process at *P* =150 W and *u* = 1000 mm/s, 750 mm/s. On the Ti_6_Al_4_V powder bed (**a**) at the ending of the first scanning track at *t* = 0.01 s, (**b**) at the ending of the final scanning track (after scanning a total of 201 tracks) at *t* = 2.01 s, (**c**) at the ending of the first scanning track at *t* = 0.01333s, (**d**) at the ending of the final scanning track (after scanning a total of 201 tracks) at *t* = 2.6793s.

The development of peak temperature with respect to time for different process parameters during the SLM process is presented in Figure 8 and Figure 9. Once the laser scan speed decreased from 1000 mm/s to 750 mm/s (at *P* = 120 W), a maximum temperature of 2306.4 K was predicted at a time of 0.0133 s, which is larger than the liquidus temperature of Ti_6_Al_4_V, as presented in Figure 8a. When the heat source reached at the final scanning track (at *t* = 2.6793 s), the observed temperature was 2624.7 K (Figure 8b), which was also over the liquidus temperature of Ti_6_Al_4_V. Once the laser power was further raised to 150 W (at *u* = 1000 mm/s), the predicted peak temperature was 2271.2 K (at *t* = 0.01 s), and it increased to 2530.1 K at the ending of the final scanning track (at *t* = 2.01 s), as demonstrated in Figure 9a,b, respectively.

Throughout the SLM process, an elevated temperature gradient can be found among the melt pool when applying higher laser power (150 W) with a comparative lower scanning speed (750 mm/s) and this phenomenon happens owing to absorption of adequate laser energy by the supplied material powders. Furthermore, an excessive heat growth phenomenon can happen which can remelt the previously built scanning path. In this situation, a higher temperature gradient of 2509.6 K (at *t* = 0.01333 s) was obtained in the melt pool which was further raised to 2891.2 K at the final point of the scanning process (at *t* = 2.6793 s), as presented in Figure 9c,d, respectively.

The numerical findings indicated that the applied laser power directly impacted the temperature fields of the powder bed throughout the SLM process; however, the laser scanning speed changed the temperature distribution by varying the laser exposure time among the applied heat source and the powder bed.

### 5.3. Molten Pool Dimensions

**Figure 10:**
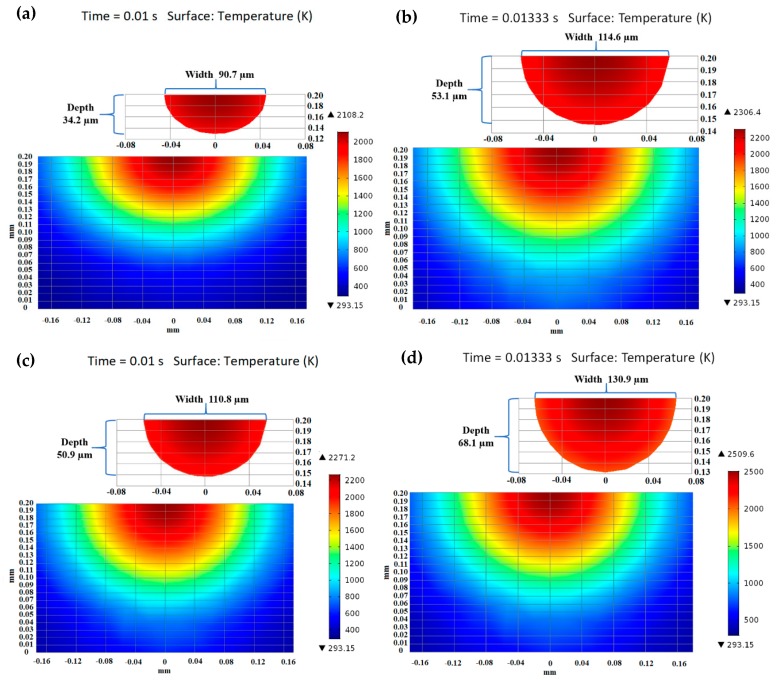
Variation in the melt pool geometry at various laser powers and scan speeds. Predicted melt pool width and depth at (**a**) 120 W-1000 mm/s, (**b**) 120 W-750 mm/s, (**c**) 150 W-1000 mm/s, (**d**) 150 W-750 mm/s, respectively.

Throughout the SLM process, it is a significant challenge to analyze the melting pool length and depth using experiments. Dilip et al. [3] experimentally determined the single-track melt pool width and depth by varying the SLM process parameters. It was reported that single-track with the process parameter sets of 150 W-750 mm/s, and 150 W-1000 mm/s had a modest energy input and produced a regular melt pool size with enough depth of penetration. The experimentally measured melt pool width and depth were approximately 134 μm and 72 μm, respectively, for the combination of 150 W-750 mm/s, and approximately 116 μm and 54 μm, respectively, for the combination of 150 W-1000 mm/s. Therefore, the calculated melt pool width and depth are comparable with the experimental findings for the single-track result. Figure 10 describes the calculated melt pool dimensions using laser powers of 120 W, and 150 W, and scanning speeds of 750 mm/s, and 1000 mm/s for the single-track laser scanning. The melting pool size (length, width, and depth) were observed from the single-track temperature distribution results considered from the melting point (1928 K) to the peak temperature along the scanning direction. At a low scanning speed, the heat source can melt the irradiated zone for a longer time rather than the high scanning speed, resulting in a large melt pool size for the high temperature in the melting zone. However, the heat source can melt the irradiated zone for a shorter time at a high scanning speed, resulting in a small melt pool for the low temperature gradient in the melting zone. Figure 10a,b clearly illustrates the calculated melt pool size for the combination of 120 W-1000 mm/s and 120 W-750 mm/s. At the same laser power of 120 W, the calculated melt pool width and depth was approximately 90.7 μm and 34.2 μm at *u* = 1000 mm/s, while the calculated melt pool depth and width was approximately 114.6 μm and 53.1 μm at *u* = 750 mm/s. Figure 10c represents the calculated melt pool size for the combination of 150 W-1000 mm/s. The calculated melt pool width was approximately 110.8 μm, which agrees with the reported experimental results with 5% error. The predicted melt pool depth was approximately 50.9 μm, which coincides with the reported experimental results with 6% error. The melt pool size is also predicted by decreasing the scanning speed to 750 mm/s at the same laser power, which is demonstrated in Figure 10d. The predicted melt pool width was approximately 130.9 μm, which was 2% less than the experimental findings, and the predicted melt pool depth was approximately 68.1 μm, which was 5% less than the reported experimental results.

**Figure 11:**
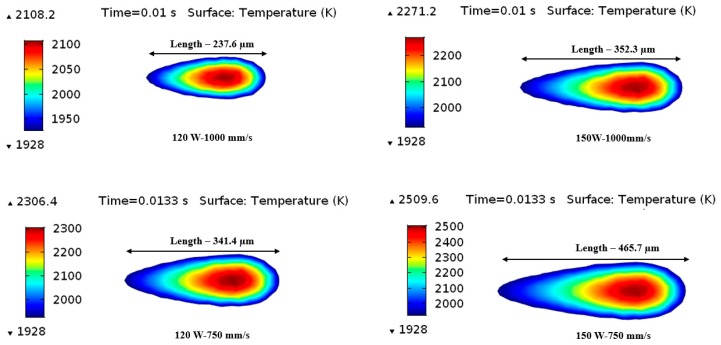
Variation of the melt pool length at different process parameters in the scanning direction at the end of the first scanning track.

Figure 11 was presented to analyze the variation of melt pool length by applying the different SLM process parameters. For each specific laser power, the melting pool length was predicted by varying the scanning speed. At a laser power of 120 W, a clear increasing tendency was determined for the melt pool length from 237.5 μm (at *u* = 1000 mm/s) to 350.7 μm (at *u* = 750 mm/s). Once the applied laser power was raised from 120 W to 150 W, the length of the melt pool improved to 450.7 μm (at *u* = 1000 mm/s) and 520.2 μm (at *u* = 750 mm/s). Therefore, the simulation findings exhibited that the melt pool size (length, width, and depth) raised linearly with the applied laser power. As can be seen from the evaluations made based on the literature, the presented numerical model can determine the melting pool width and depth in an acceptable range.

### 5.4. Experimental Validation

**Figure 12:**
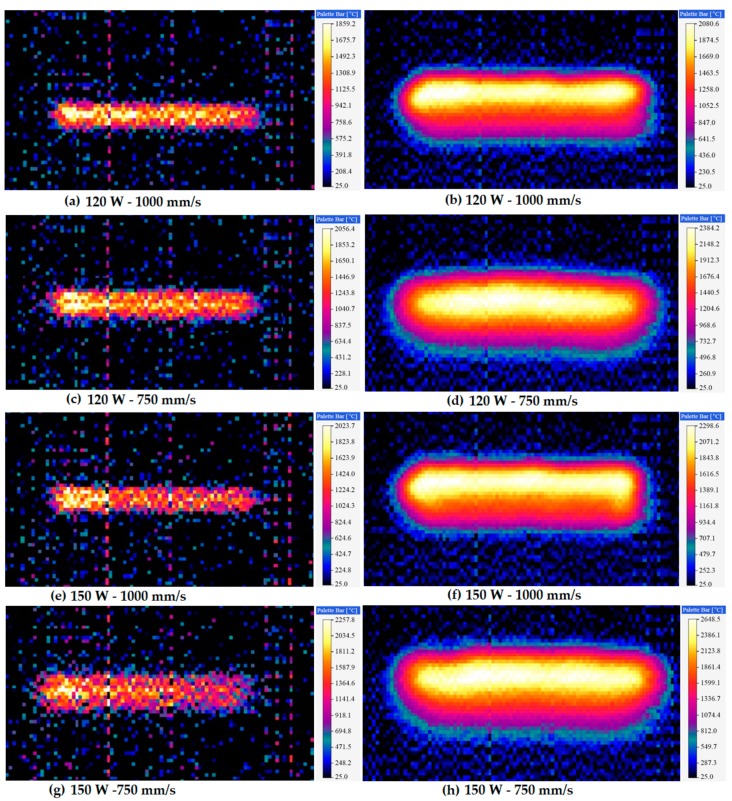
Typical thermal images for different laser powers and scanning speeds along the scanning direction. (**a**) Temperature gradient at a time of 0.01s for 120 W-1000 mm/s. (**b**) Temperature profiles at a time of 2.01s (after scanning a total of 201 tracks) for 120 W-1000 mm/s. (**c**) Temperature profiles at the time of 0.01333s for 120 W-750 mm/s. (**d**) Temperature profiles at a time of 2.6793s (after scanning a total of 201 tracks) for 120 W-750 mm/s. (**e**) Temperature profiles at a time of 0.01s for 150 W-1000 mm/s. (**f**) Temperature profiles at a time of 2.01s (after scanning a total of 201 tracks) for 150 W-1000 mm/s. (**g**) Temperature profiles at a time of 0.01333s for 150 W-750 mm/s. (**h**) Temperature profiles at a time of 2.6793s (after scanning a total of 201 tracks) for 150 W-750 mm/s.

To confirm the reliability of the presented simulation model, experiments were carried out to determine the temperature distributing characteristics throughout the SLM process. By using the thermal imager, the developed temperature profiles in the melt pool were monitored, and the images were captured in real time. Figure 12 displays the characteristics of temperature distributions and melt pools at various laser powers and scanning speeds. To measure the temperature, thermal camera emissivity was fixed to 0.35 with a transmission rate of 1.0. These thermal pictures represent the temperature profiles obtained by moving the heat source. A high-temperature region was found close to the laser spot center and progressively cooled down behind the melting pool as the heat source moved away. Figure 12a presents the temperature profiles for the first scanning track at *P* = 120 W and *u* = 1000 mm/s. By means of the heat source moved through the powder bed in the first scanning track, the temperature increased rapidly and exceeded the melting point, leading to powder melting. The temperature progressively decreased as the laser beam moved away. Taking the emissivity as ε~0.35, the highest temperature of the melt pool at the time of 0.001s was about 2132.3 K (1859.2 °C) (white color) which surpassed the melting temperature of Ti_6_Al_4_V. For multi-track scanning, the hatch distance influences the highest temperature in the present scanning track due to the enduring heat provided by the previous tracks. Since the radius of the laser beam is larger than the hatch distance of 30 μm, some areas of the former track remelted, which led to the rise of the peak temperature at the ending of the SLM process. As a result, the temperature increased to 2353.7 K (2080.6 °C) at the end point of the final track, and the total scanning time was 2.01 s, as presented in Figure 12b. In case of low scanning speed of 750 mm/s (at the same *P* = 120 W), the temperature increased to 2329.5 K (2056.4 °C) at a time of 0.01333 s, and 2657.3 K (2384.2 °C) at a time of 2.6793 s, as presented in Figure 12c,d, respectively.

Another set of experiments was carried out at the process parameter sets of 150 W-1000 mm/s and 150 W-750 mm/s. Figure 12e describes the temperature contours at the end of the first scanning track for *P* = 150 W and *u* = 1000 mm/s. The peak temperature in the melt pool was determined approximately 2296.8 K (2023.7 °C) at 0.01 s. The temperature increases in the second track leading to the end track were attributed to reheating induced by the heat source due to hatch spacing, and the peak temperature was reached to 2571.7 K (2298.6 °C) at the end of final scanning track, as shown in Figure 12f. Figure 12g further demonstrates that the highest temperature of the melt pool increased to 2527.4 K (2257.8 °C) at the time of 0.01333 s, as the scanning speed decreases to 750 mm/s (at the same *P* = 150 W). The reason behind this peak temperature is the tremendous energy density provided by the heat source. Furthermore, the highest temperature measured by the thermal imager was about 2891.2 K (2648.5 °C) at a time of 2.6793 s.

**Figure 13:**
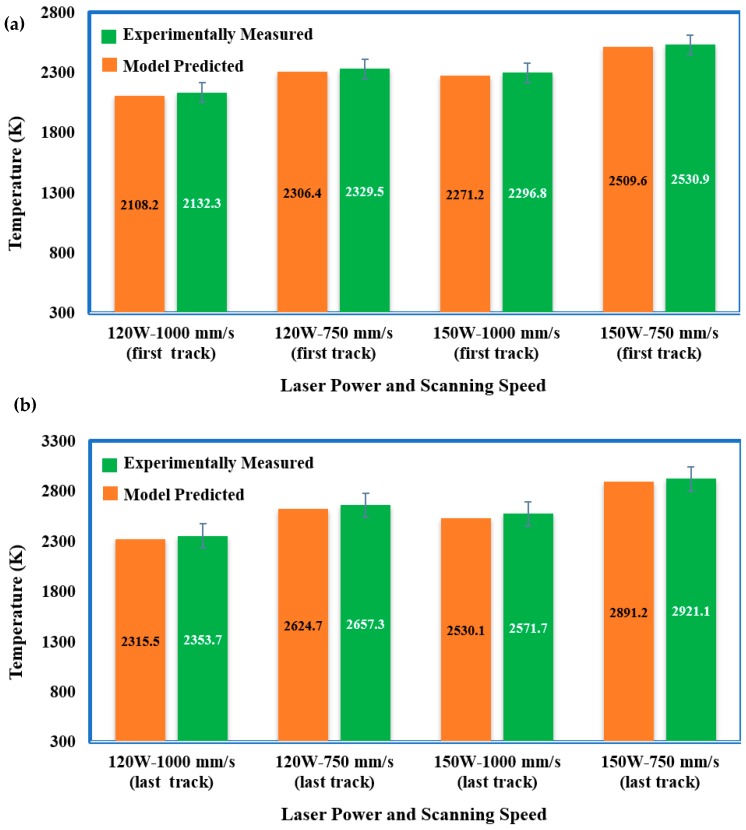
Comparison of experimental and model-predicted peak temperature distribution results: (**a**) at the ending of the first track and (**b**) at the ending of the final scanning track.

Figure 13 demonstrates a comparison of the numerically predicted and experimentally measured peak temperature distribution results. The developed numerical model presented a reasonably precise prediction based on a comparison with the experimentally measured peak temperature. For the temperature measurement, the predicted errors among the simulation and experimental results are in a range of 21–40 K. These errors can occur because of the scattering in the experimental results and probable differences between the thermo-physical properties used for the numerical analysis and real properties in the experiments. Overall, the developed model is appropriate to employ in the thermal modeling and numerical analysis for the SLM process of Ti_6_Al_4_V powder.

## 6. Conclusions

In this study, a 3D FE model was established to evaluate and predict the temperature distribution and the melt pool size during the SLM process. Furthermore, a thermal imager was used to determine the temperature gradients of the Ti_6_Al_4_V material employing the same process parameters as those used in the numerical investigation. The major conclusions of this study can be summarized as follows:

1. The thermal imager was able to capture the temperature profiles at various laser powers and scanning speeds under the same camera setting, e.g., emissivity = 0.35 and transmission rate = 1.0. Besides, the developed model correctly determined the temperature distribution along the laser scanning direction with good correlation to the experimentally measured temperature for both the single-track and multi-track scanning. The predicting error of the established model is in the range of 21–40 K.
2. At a laser power of 150 W, the predicted melt pool width and depth were approximately 130.9 μm and 68.1 μm, respectively for the 750 mm/s scanning speed; while the length and width would be 114.6 μm and 53.1 μm, respectively for a scanning speed of 1000 mm/s. The developed model can predict the melt pool width (with 2–5% error) and melt pool depth (with 5–6% error).
3. Therefore, the presented fluid flow model that includes the heat flow behavior among the melt pool owing to Marangoni convection is an effective technique for modeling theTi_6_Al_4_V powder melting behavior. Furthermore, the peak temperature, and the length, width, depth of the melt pool all rises as the laser power rises and the scanning speed decreases.
4. The calculation of peak temperature and melt pool size for the single-track depositions would be an important method for exploring the optimal process parameters for the SLM process. From the above investigation, SLM process parameters with the sets of 150 W-750 mm/s result in enough temperature to melt the powder, provide a well-defined melt pool size and create a uniform single track. Therefore, this set of process parameters would be suggested for manufacturing 3D printed parts by using the SLM process for the Ti_6_Al_4_V alloy.

Furthermore, the established thermal model can be used to explore the temperature fields and melt pool size for other materials. It could also be expanded to 3D freeform complex geometries manufactured with the SLM process.

## Figures and Tables

**Table 1:** Process parameters and thermo-physical properties of Ti_6_Al_4_V [20,21] used in this finite element simulation.

Name	Description	Value
P	Laser power (W)	120, 150
u	Laser scanning speed (mm/s)	750, 1000
R	Laser spot radius (μm)	50
A	Absorption coefficient	0.3
δ	Optical penetration depth (μm)	65
h	Hatch distance (μm)	30
ε	Emissivity	0.35
σ	Stefan–Boltzmann constant (W/ (mm^2^·K))	5.67 × 10^−14^
µ	Dynamic viscosity (Pa·s)	0.002
L_f_	Melting latent heat (J·kg^−1^)	3.5 × 10^5^
dγ/dT	Surface tension gradient (N·m^−1^·K^−1^)	−2.7 × 10^−4^
T_L_	Liquidus temperature (K)	1928
T_S_	Solidus temperature (K)	1878
